# Effects of iron oxide contents on photocatalytic performance of nanocomposites based on g-C_3_N_4_

**DOI:** 10.1038/s41598-023-33338-1

**Published:** 2023-04-17

**Authors:** M. Afkari, S. M. Masoudpanah, M. Hasheminiasari, S. Alamolhoda

**Affiliations:** School of Metallurgy and Materials Engineering, Iran University of Science and Technology (IUST), Tehran, Iran

**Keywords:** Materials science, Nanoscience and technology

## Abstract

α-Fe_2_O_3_/Fe_3_O_4_/g-C_3_N_4_ nanocomposites were prepared in-situ by solution combustion as magnetically separable photocatalysts using ferric nitrate as oxidant, glycine as organic fuel, and g-C_3_N_4_. The effects of various amounts of iron oxides, on the magnetic, optical, and photocatalytic properties were explored by different characterization methods. The magnetite (Fe_3_O_4_) phase as ferrimagnetic material disappeared with the increase in ferric nitrate contents, leading to the decrease of magnetic properties. The bandgap energy decreased from 2.8 to 1.6 eV with the increase of the hematite (α-Fe_2_O_3_) phase.The photocatalytic results showed that the type and amount of iron oxides had a significant effect on the decolorization of methylene blue, rhodamine B and methyl orange dyes under visible-light irradiation. The activity of the nanocomposite sample containing 37 wt. % iron oxides was more effective than that of the pristine g-C_3_N_4_ sample to photodegrade the methylene blue, rhodamine B and methyl orange, respectively. Moreover, the nanocomposites exhibited a higher photocurrent density than that of the pristine g-C_3_N_4_, mainly due to their lower charge recombination rate.

## Introduction

Semiconductor photocatalysts have been the subject of significant attention as a simple time and energy-efficient technology to convert organic pollutants into eco-friendly mineralized byproducts.under solar irradiation^1^. Several versatile semiconductor photocatalysts, including transition metal oxides, hydroxides, sulfides, phosphates and transition metal dichalcogenides of various particle sizes and shapes and also two dimensional (2D) materials were widely applied for wastewater treatment by degrading organic dyes under sunlight^2–5^.But the application of photocatalysts suffers from poor photodegradation efficiency, lower chemical stability, and poor exploitation of the solar spectra. Therefore, many efforts have been made to find a photocatalyst system without these shortcomings^6,7^.

Graphitic carbon nitride (g-C_3_N_4_) has been considered a metal-free semiconductor for water splitting, photodegradation of toxic organic pollutants, CO_2_ reduction and antibacterial agents to disinfect the antibiotic-resistant microorganism strains^8,9^. The non-toxicity, low cost, narrow bandgap (Eg≈2.7 eV), and high chemical stability are unique features of 2D polymeric g-C_3_N_4_ material^8,10^. Furthermore, the g-C_3_N_4_ material can be easily synthesized by one-step polycondensation of organic materials such as urea, thiourea, melamine, cyanamid, and dicyandiamide containing nitrogen atoms^11,12^. However, the high recombination rate, low visible-light absorption coefficient and secondary pollution are three main limitations for efficient applications of g-C_3_N_4_ material on an industrial scale^13^. To address the disadvantages, the g-C_3_N_4_ material can be modified by other materials such as TiO_2_, ZnO, WO_3_ and α-Fe_2_O_3_ to extend the visible-light absorption capacity and separation of photogenerated electron–hole pairs that prevents the fast electron–hole recombination reaction^14–17^. Furthermore, magnetic materials such as Fe_3_O_4_, ZnFe_2_O_4_, CoFe_2_O_4_, and BiFeO_3_ can be loaded on the pristine g-C_3_N_4_ material to form a magnetically separable photocatalyst^18–21^.

Among various materials, iron oxides such as α-Fe_2_O_3_, γ-Fe_2_O_3_, and Fe_3_O_4_ with chemical and thermal stability, moderate magnetic properties, and optical properties have been widely used in several magnetic, catalyst, and photocatalyst applications because of their ease of synthesis, abundance, low cost, and environmentally benign^22–24^. Therefore, the iron oxides can be suitable candidates to combine with g-C_3_N_4_ material to improve the photocatalytic performance via enhancing visible-light absorbance, separation of the charge carriers, and magnetic recyclability^25–27^. For example,α-Fe_2_O_3_ has high absorption (~ 43%) in the red region of visible light, making it a proper candidate for coupling with g-C_3_N_4_ for photodegradation^28,29^. Ghane et al.^30^ reported the in-situ preparation of g-C_3_N_4_/α-Fe_2_O_3_ nanocomposite by solution combustion synthesis method for decolorization of methylene blue (MB) dye under visible light irradiation. They optimized the amount of α-Fe_2_O_3_ phase to obtain high photodegradation and photocurrent density. However, the α-Fe_2_O_3_ phase has antiferromagnetic behavior with negligible magnetic properties, which are insufficient for recycling the g-C_3_N_4_/α-Fe_2_O_3_.

It was observed that high conductivity and suitable energy band structure of Fe_3_O_4_ can enhance the photocatalytic performance of composite photocatalysts due to the separation of electron − hole pairs^31–33^. Mousavi and Habibi-Yangjeh also reported the effective role of bismuth oxyiodide (BiOI) species on the photodegradation of rhodamine B (RhB) by g-C_3_N_4_/Fe_3_O_4_ nanocomposites^34^. The BiOIphase was used for electron trapping, while the magnetic recycling was obtained by the Fe_3_O_4_ phase^34^. The Fe_2_O_3_/Fe_3_O_4_/g-C_3_N_4_ nanocomposite was previously synthesized by the hydrothermal method^31^. Up to our knowledge, it was not prepared by the simple and cost-effective solution combustion route in which the amounts of various iron oxides can be easily changed by tuning the synthesis conditions.

In this work, the solution combustion method was applied for the in-situ preparation of α-Fe_2_O_3_/Fe_3_O_4_/g-C_3_N_4_ nanocomposites with the various amounts of α-Fe_2_O_3_ and Fe_3_O_4_ phases. The nanocomposites' structural, microstructural, and photoelectrochemical properties were characterized by different techniques including X-ray diffractometry, thermogravimetry, Raman spectroscopy, electron microscopy, N_2_ adsorption–desorption isotherms, vibrating sample magnetometry, diffuse reflectance spectroscopy, photoluminescence spectroscopy, dye photodegradation, electrochemical impedance spectroscopy, and photoelectrochemical tests.

## Experimental procedures

Analytical grade of ferric nitrate (Fe(NO_3_)_3_.9H_2_O, ≥ 98%), glycine (C_2_H_5_NO_2_, ≥ 99%), and melamine (C_3_H_6_N_6_, ≥ 99%) were purchased from Merck company.

### Synthesis of α-Fe_2_O_3_/Fe_3_O_4_/g-C_3_N_4_nanocomposites

The g-C_3_N_4_powder was prepared by heating 10 g melamine powder from room temperature up to 550 °C at a rate of 10 °C/min in a muffle furnace. After holding at 550 °C for 3 h, the products were cooled in the furnace^8^.

The α-Fe_2_O_3_/Fe_3_O_4_/g-C_3_N_4_ nanocomposites were prepared as follows: 2 g graphitic carbon nitride powder was dispersed into 30 mL distilled water. The ferric nitrate as oxidant and glycine as fuel were added to the suspension. The amounts of Fe(NO_3_)_3_.9H_2_O were adjusted to form 0.05, 0.10, and 0.20 g iron oxides in the final products which were related to 2.5 wt. %, 5 wt. %, and 10 wt. %, respectively. The molar ratio of glycine fuel to Fe(NO_3_)_3_.9H_2_O was set to 1.67^35^. The precursor solution was stirred and evaporated at 80 °C until completely dried. The combustion reaction was started by heating the dried gel to 250 °C on a hotplate. For easy presentation, the nanocomposites were coded on the base of intended iron oxide contents; for example, the symbol “X5” shows that the desired mass ratio of iron oxide was 5 wt. %.

### Characterization methods

The structure and phase of the combusted products were characterized by the X-ray diffractometry (XRD) technique. The diffraction patterns were collected on a D8 ADVANCE (Bruker, Kanagawa, Japan) using Cu Kα radiation (λ = 1.5418 A°). The combusted powders were thermally analyzed on an STA 503 instrument (Bahr, Germany) with a heating rate of 10 °C/min in the air atmosphere. Raman spectra were recorded on TEKSAN N1-541 spectrophotometer with Nd:YAG laser source. The saturation magnetization of the combusted powders was obtained from hysteresis loops measured on a vibrating sample magnetometer (MeghnatisKavir Kashan Co., Iran). The particle size and elemental distribution were obtained on MIRA3(TESCAN, Czech Republic) scanning and CM200 (Philips, UK) transmission electron microscopy. The specific surface areas, pore size distribution, and pore volume were calculated from the N_2_ adsorption/desorption isotherms measured on PHSCHINA (PHS-1020, China) instrument. The bandgap energy was obtained from diffuse reflectance spectra recorded on a 52550 UV–Vis (Shimadzu, Japan) spectrophotometer. The charge separation was studied by photoluminescence (PL) spectra which were obtained on a G9800A (Agilent, USA) fluorescence spectrophotometer (λ_Ex._ = 320 nm).

### Photocatalytic test

In order to evaluate the photocatalytic performance of the nanocomposite for positively and negatively-charged dyes, 5 ppm solution of methylene blue (MB), rhodamine B (RhB) and methyl orange (MO) dyes were photodegraded by 0.1 g samples under irradiation of two 100 W Xenon lamps with the intensity of 1000 $\frac{mW}{{cm}^{2}}$ as visible light source. The UV light was filtered by a cutoff filter (λ = 420 nm). Before turning on the light, the solution was stirred in dark for 1 h to establish the adsorption/desorption equilibrium of the dye. A 2600 UV–Vis spectrophotometer (Shimadzu, Japan) was used to monitor the relative concentrations of the dyes versus time.

### Photoelectrochemical (PEC) test

The working electrode was prepared by dispersing 0.01 g nanocomposites in 0.5 mL NMP solution containing PVDF by sonication. Then the slurry was coated on 2 × 2 cm^2^ fluorine-doped tin oxide (FTO) glass and dried in an oven overnight. The working electrode was used in a three-electrode system in which Ag/AgCl and Pt were referenceand counter electrodes, respectively. The electrolyte was an aqueous solution of Na_2_SO_4_ (0.1 M). All of the electrochemical tests including electrochemical impedance spectroscopy, linear scanning voltammetry, and chronoamperometry were carried out on an OrigaFlexOGF01SOLARERON electrochemical workstation. The 500 W Xe lamp with an IR filter was used as a light source.

## Results and discussion

### Materials characterization

**Figure 1:**
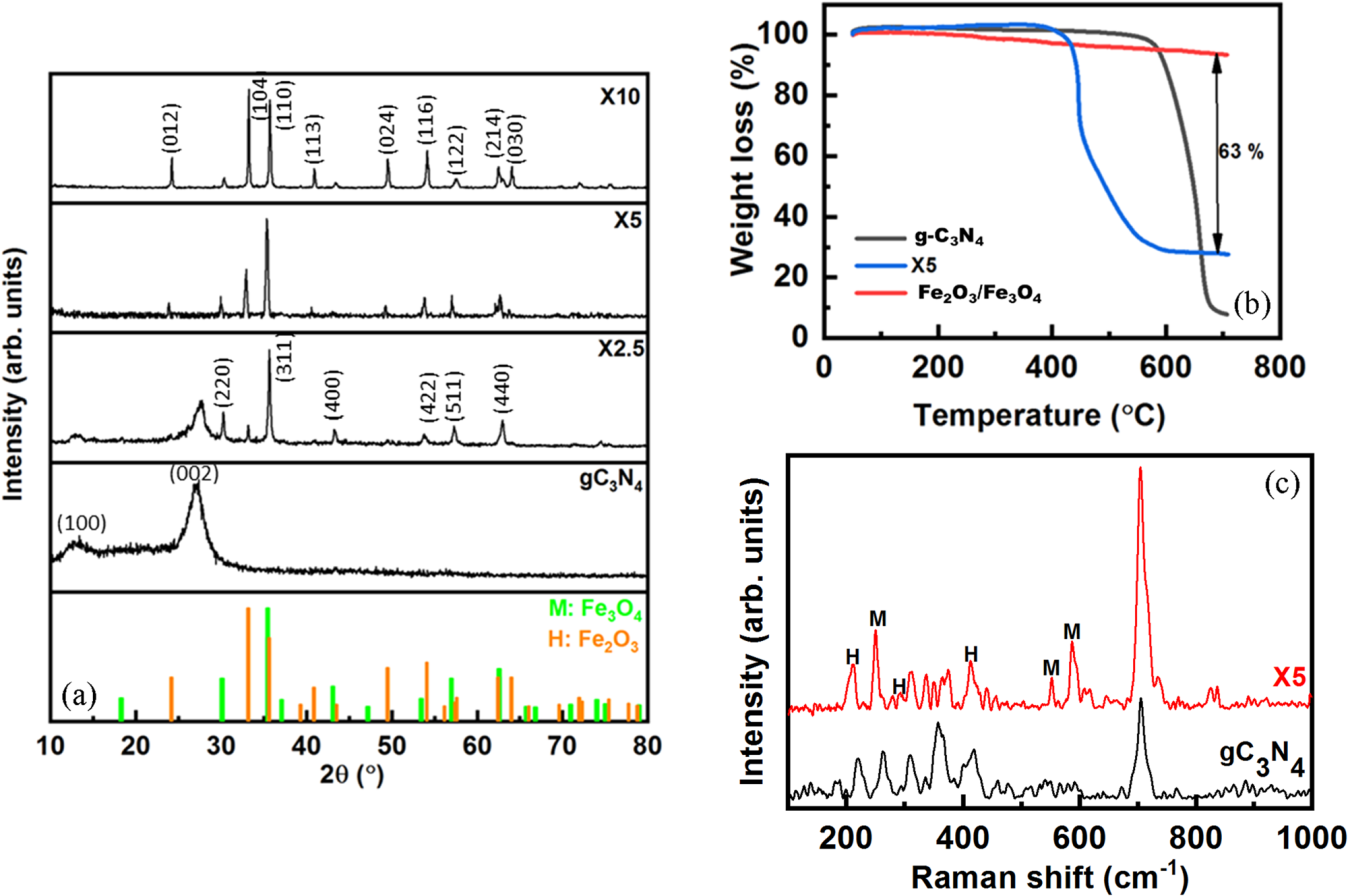
(**a**) XRD patterns, (**b**) TGA curves, and (**c**) Raman spectra of the pristine gC_3_N_4_ powders and the X2.5, X5, and X10 samples.

Figures 1a–c show the XRD patterns, Raman spectra, and TGA curves of the pristine g-C_3_N_4_ powder and nanocomposites. The two reflections at 2θ = 13.1° and 27.3° for the pristine g-C_3_N_4_ powders are related to the (100) plane of the in-planar motif of tri-s-triazine units and (002) plane of interlayer stacking of conjugated carbonaceous rings, respectively^27^. In addition to the reflections of the g-C_3_N_4,_ the X2.5 sample shows mainly the diffraction peaks of the Fe_3_O_4_ phase (PDF2#00-033-0664) accompanied by a few amounts of α-Fe_2_O_3_ phase (PDF2#00-033-0664). The diffraction peaks of the α-Fe_2_O_3_ phase are intensified, while those of the Fe_3_O_4_ phase are weakened in the X5 and X10 samples. The X10 sample has a higher amount of α-Fe_2_O_3_ phase and some of the Fe_3_O_4_ phase. However, the (100) and (002) reflections of the g-C_3_N_4_ phase are absent in the X5 and X10 samples. The solution combustion synthesis (SCS) involves an exothermic reaction between ferric nitrate as oxidant and glycine as fuel. The combustion reaction begins by endothermal decomposition of ferric nitrate to α-Fe_2_O_3_ as solid products and O_2_ and N_2_ as gaseous products as follows^36,37^:

1 $$ {\text{4Fe}}\left( {{\text{NO}}_{{3}} } \right)_{{3}} \to {2}({{\alpha}} - {\text{Fe}}_{{2}} {\text{O}}_{{3}} ) + {\text{15O}}_{{2}} + {\text{6N}}_{{2}} $$

The glycine molecules can be exothermally oxidized to CO_2_, N_2_, and H_2_O gases by the liberated O_2_ gas as follows^38^:

2 $$ {\text{2C}}_{{2}} {\text{H}}_{{5}} {\text{NO}}_{{2}} + {\text{9O}}_{{2}} \to {\text{4CO}}_{{2}} + {\text{N}}_{{2}} + {\text{5H}}_{{2}} {\text{O}} $$

Reaction (2) releases higher thermal energy than that of the required heat to proceed with the reaction (1), leading to the self-propagating nature of the SCS^39^. Moreover, the liberated gases can reduce, oxidize, and sulfidize the solid products^40^. Therefore, the appearance of the Fe_3_O_4_ phase instead of the α-Fe_2_O_3_ phase in the X2.5 sample can be attributed to the reduction of α-Fe_2_O_3_ phase by reductive H_2_ and CO gases. However, the higher combustion temperature in the X5 and X10 samples prevents the effective reduction of α-Fe_2_O_3_ phase to Fe_3_O_4_ phase. Furthermore, the absence of g-C_3_N_4_ peaks in the X5 and X10 samples can be attributed to the stacking disorders by introducing a mixture of α-Fe_2_O_3_/Fe_3_O_4_ phases^41,42^. Figure 1b shows TGA curves of the X0, X5, and X100 powders to calculate the amount of loaded α-Fe_2_O_3_/Fe_3_O_4_ nanoparticles on g-C_3_N_4_. The g-C_3_N_4_ starts to decompose at 600 °C, which can be completed at higher temperatures (> 700 °C) by burning g-C_3_N_4_^43,44^. The α-Fe_2_O_3_/Fe_3_O_4_ powder shows a low weight loss of 7%, while the X5 composite has a higher weight loss of 73% due to the burning of g-C_3_N_4_. Therefore, the residual fraction of the X5 sample is 37 wt. %. Furthermore, the g-C_3_N_4_/α-Fe_2_O_3_/Fe_3_O_4_ nanocomposite begins to lose its weight at the lower temperature of 400 °C, which can be attributed to the higher induced crystal defects in g-C_3_N_4_ species by combustion reaction. The higher amount of solid product of the as-combusted X5 sample can be attributed to the burning of g-C_3_N_4_ during combustion temperature. Figure 1c shows Raman spectra of the g-C_3_N_4_ and X5 samples. The characteristic Raman bands of the g-C_3_N_4_ phase can be identified at 220, 262, 311, 350, 480, and 708 cm^−1^^45^. The X5 sample shows the bands at 221, ~ 294, ~ 410, ~ 498, and ~ 607 cm^−1^ for the α-Fe_2_O_3_ phase and 370, 520, and 670 cm^−1^ for the Fe_3_O_4_ phase^46^, in addition to the bands related to the g-C_3_N_4_ phase. The most intense peak at 708 cm^−1^ corresponding to the s-triazine ring confirms the existence of g-C_3_N_4_ despite its absence in XRD patterns (Fig. 1a).

The magnetization curves of the X2.5 and X5 samples and the separation of the composite from solution by a magnet are presented in Fig. S1 (supplementary information). The ferromagnetic behavior of the composite powders is due to the presence of the ferrimagnetic Fe_3_O_4_ phase. By the appearance of the antiferromagnetic α-Fe_2_O_3_ phase, the saturation magnetization (M_s_) decreases from 22 to 18 emu/g. Furthermore, the lower Ms value of the X2.5 sample than that of bulk Fe_3_O_4_ can be attributed to the nonmagnetic g-C_3_N_4_. The magnetic properties of the composite are enough for recycling the photocatalyst following the decontamination processes. Wang et al.^31^ synthesized a similar composite by hydrothermal method. The Ms value of the α-Fe_2_O_3_/Fe_3_O_4_/g-C_3_N_4_ nanocomposite is in the same range as this work (17.98 emu/g). Furthermore, the high magnetic properties confirm the existence of Fe_3_O_4_ as ferrimagnetic phase, because the α-Fe_2_O_3_ phase is antiferromagnetic material with very low saturation magnetization.

**Figure 2:**
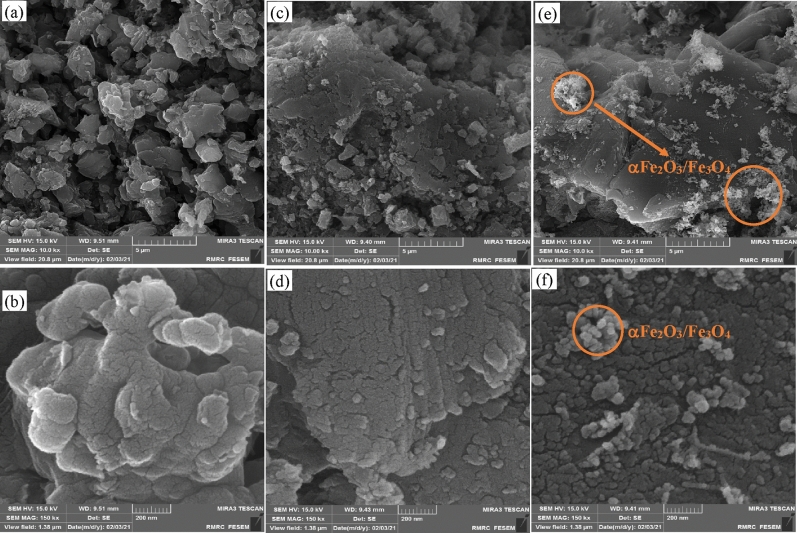
SEM and images of (**a** and **b**) g-C_3_N_4_, (**c** and **d**) X2.5, and (**e** and** f**) X5 powders.

**Figure 3:**
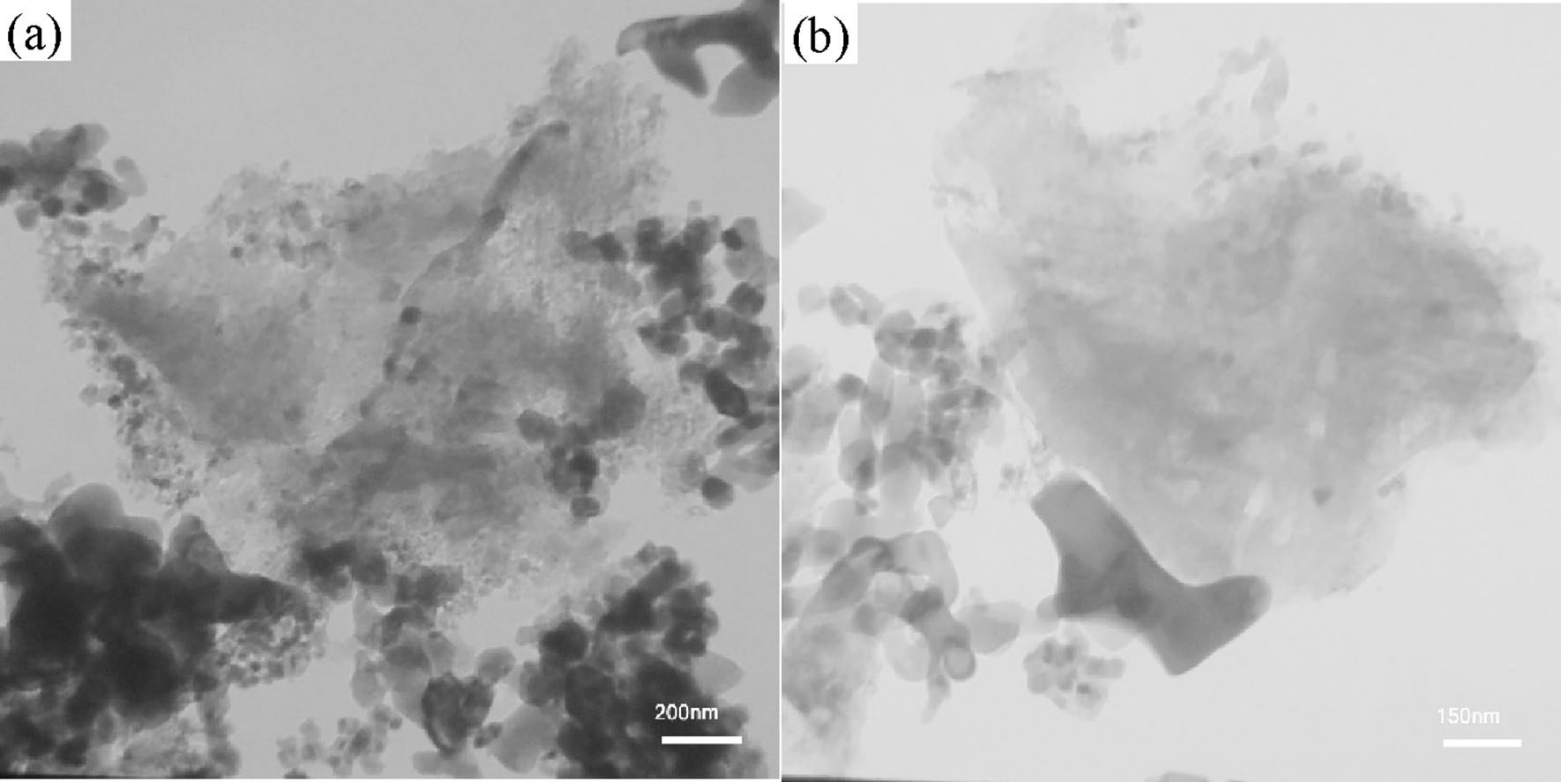
TEM images of the X5 composite powders.

Figure 2 shows the FESEM images of the pristine g-C_3_N_4_ powder and X2.5 and X5 nanocomposites. The pure g-C_3_N_4_ powders are composed of secondary particles (1–3 μm) (Fig. 2a) with small primary particles of multiple wrinkled-layer-stack of g-C_3_N_4_ (Fig. 2b). After impregnating g-C_3_N_4_ powders with the precursor solution of ferric nitrate and rendering the combustion reaction, the nanoparticles of iron oxides are distributed on the edge and surface of g-C_3_N_4_ powders (Fig. 2c,d). With the increase of the amount of nanoparticles of iron oxides, the nanoparticles are aggregated into larger particles (Fig. 2e,f). The non-uniform distribution of the nanoparticles of iron oxides can be attributed to the hydrophobic behavior of the surface of g-C_3_N_4_^47^. TEM images of the X5 composite powders are given in Fig. 3a–b. The particles of iron oxides are aggregated on the edge and surface of g-C_3_N_4_ powders. Furthermore, there are some pores between iron oxide nanoparticles.

In SCS, for the synthesis of g-C_3_N_4_/α-Fe_2_O_3_/Fe_3_O_4_, the g-C_3_N_4_ particles were dispersed in a precursor solution containing the ferric nitrate and glycine. The precursor solution was dried by heating up at 80 °C, leading to the formation of gel. Therefore, if the surface of g-C_3_N_4_ had hydrophilic nature, a thin and continuous film of gel can be easily formed on it which transformed into well-dispersed nanoparticles during the combustion reaction. The non-uniform distribution of the nanoparticles of iron oxides on the surface of g-C_3_N_4_ may be caused by the hydrophobic behavior of precursor solution. It is worth noting that the iron oxide particles have a quasi-spherical shape. Elemental distributions of the C, O, and Fe and the superposition of elemental distribution of Fe and C in the region shown in Fig. S2a are given in Figs. S2b–e, respectively. There is C element over all regions, while the Fe element is concentrated on the brighter aggregate.

N_2_ adsorption–desorption isotherms and pore size distribution plots of the X2.5 and X5 composite powders are presented in Fig. S3. The isotherms are IV type with H3 hysteresis loop related to the mesoporous network according to the IUPAC classification^48^. The X5 composite has a higher specific surface area (32 m^2^/g) and pore volume (0.2 cm^3^/g) than those of the X2.5 sample (19 m^2^/g and 0.1 cm^3^/g). The textural properties of the combusted products are a compromise of the amount of liberated gases and heat in which the lower released heat and higher exhausted gases lead to higher specific surface areas, higher pore volume, and larger pores due to the suppression of particles’ sintering and disintegration of large particles^49^. Therefore, the higher specific surface of the X5 composite sample can be s ascribed to the release of higher liberated gaseous products. The mesoporous nature of the composite samples provides further reaction sites, which improves the photocatalytic efficiency via charge separation.

It is well-known that photodegradation performance strictly depends on the light absorption coefficient. The UV–Vis diffuse reflectance spectroscopy was applied to show the photoabsorption ability of the composite samples (Fig. S4a). The pristine g-C_3_N_4_ powders have an absorption edge of about 470 nm, leading to a negligible absorption in the visible range. However, the ability of the composite samples is considerably higher for light absorption in the visible range. Furthermore, the light absorption in the visible range increases with the increase of α-Fe_2_O_3_ content, appropriating the application of α-Fe_2_O_3_/Fe_3_O_4_/g-C_3_N_4_ nanocomposites as visible-light-driven photocatalysts. According to Taucʼs equation, the bandgap energy (Eg) can be estimated as follows^50^:

3 $$ \left( {\alpha {\text{h}}\nu } \right)^{{{2}/{\text{n}}}} = {\text{A}}({\text{h}}\nu - {\text{E}}_{{\text{g}}} ) $$

where α is the light absorption coefficient, and the n value is related to the transition type^51^. Therefore, the bandgap energy (Eg) can be obtained by extrapolating the linear part of the (αhν)^2^ curves versus hν to 0 (Fig. S4b). The E_g_ values of g-C_3_N_4_, X2.5, X5, and X10 samples are 2.8, 2.5, 1.6, and 2 eV, respectively. The bandgap of the composite samples lies between the pristine g-C3N4 and pristine α-Fe2O3 powders, indicating a good interaction between both components, resulting in the bandgap alignment^52^. The very narrow bandgap of the X5 sample can be attributed to the more heterogeneous distribution of the α-Fe_2_O_3_ phase.

### Photocatalytic performance

**Figure 4:**
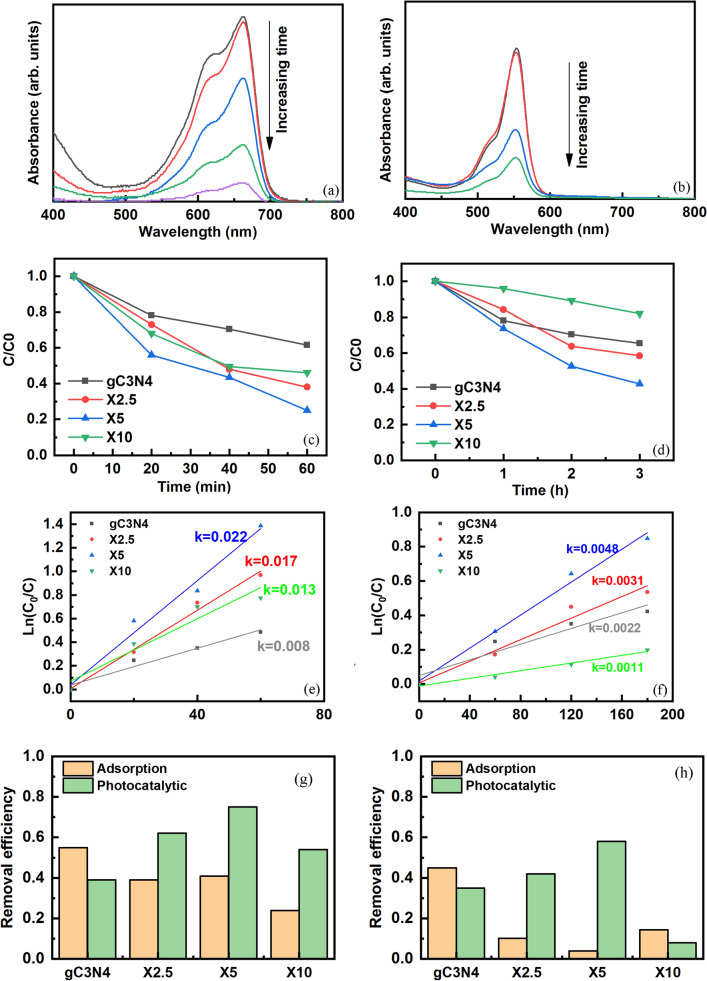
UV–Vis spectra of (**a**) MB and (**b**) RhB dye in the presence of the X5 composite sample, the relative concentration of (**c**) MB and (**d**) RhB dyes versus illumination time, Ln(C0/C) versus time for (**e**) MB and (**f**) RhB dyes, and removal efficiency of (**g**) MB and (**h**) RhB dyes by various catalysts.

Figure 4a,b show the UV–Vis spectra of the MB and RhB solutions versus light illumination time in the presence of the X5 sample as photocatalyst. The weakening of the characteristic peaks of MB and RhB dyes at 665 and 550 nm, respectively, shows the degradation of dye structure with time. The dependence of the relative concentration of dyes versus time in the presence of g-C_3_N_4_, X2.5, X5, and X10 samples are given in Fig. 4c,d. The photodegradation rate of MB and RhB dyes can be fitted by the pseudo-first kinetics as follows:

4 $$ {\text{Ln}}\left( \frac{C}{C0} \right) = - kt $$

The values of k (min^−1^) as rate constant calculated by plotting Ln $\left(\frac{C}{C0}\right)$ versus irradiation time, as shown in Fig. 4e,f. The constant rate of photodegradation increases 2.5 and 4 times for MB and RhB dyes, respectively, by the combination of g-C_3_N_4_ with a proper amount of iron oxides. The removal efficiencies, including adsorption and photodegradation processes of MB and RhB dyes in the presence of various catalysts, are summarized in Fig. 4g,h. About 40% of MB is photodegraded by the g-C_3_N_4_ catalyst, which increases to 62 and 75 for the X2.5 and X5 powders, respectively. However, the photodegradation decreases to 54% for the X10 catalysts after 60 min of illumention. For RhB dye and following illumination for three h, the pristine g-C_3_N_4_ powders photodegrade about 35% while the amount of photodegradation firstly increases up to 58% with the addition of α-Fe_2_O_3_/Fe_3_O_4_ nanoparticles and then decreases to 8% for the X10 sample, possibly due to the disappearance of Fe_3_O_4_ as the charge acceptor. The separation of photogenerated charges, higher light absorbance, and higher specific surface areas are responsible factors for the higher photocatalytic activity of the nanocomposites^30^. Furthermore, the adsorption capacity of the MB dye is higher than that of the RhB dye because of the strong interaction between the surface ligands of the catalyst and MB dye molecules^34^.

In order to evaluate the photocatalytic performance of the nanocomposites to the negatively charged dyes, MO dye photodegradation by the composites was also tested. Fig. S4a shows the dependence of the relative concentration of methy orange (MO) in the presence of samples g-C_3_N_4_, X2.5, X5 versus time. MO degradation was not occurred in presence of sample X10. The photodegradation rate of MO was fitted by the pseudo-first kinetics (Fig. S4b). The constant rate of photodegradation increases 3 times for MO by the combination of g-C_3_N_4_ with a proper amount of iron oxides (sample X5). The removal efficiencies, including adsorption and photodegradation processes of MO dyes in the presence of various catalysts, are summarized in Fig. S5c. About 30% of MO is photodegraded by the g-C_3_N_4_ catalyst, which decreases to 14 and then increases to 53 for the X2.5 and X5 powders, respectively. This reveals that the the adsorption capacity of the MO dye is lower than the other two dyes since the electrostatic interactions of the MO (which is an anionic dye) is different with the surface of the catalyst^53^.

The photocatalytic process involves three main steps: (i) generation of electron (e^−^) and hole (h^+^) pairs by absorbing the light photons, which have higher energy than that of the bandgap energy of semiconductor; (ii) migration of charge carries without their recombination to the surface of catalyst; (iii) redox reactions between the photogenerated charges with solvated species such as O_2_ and H_2_O to produce highly oxidant agents like.O_2_ and OH on the surfaceof catalyst^54^. Therefore, the bandgap energy, absorption coefficient, and charge recombination as optical characteristics of catalyst have an effective role on the photocatalytic activity^6^. Because the g-C_3_N_4_ and α-Fe_2_O_3_ phases in the composites show different ranges of photoabsorption, their combination broadens the visible-light photoresponse and narrows the bandgap (Fig. S4), leading to the generation of a great number of charge carriers under visible light illumination.

**Figure 5:**
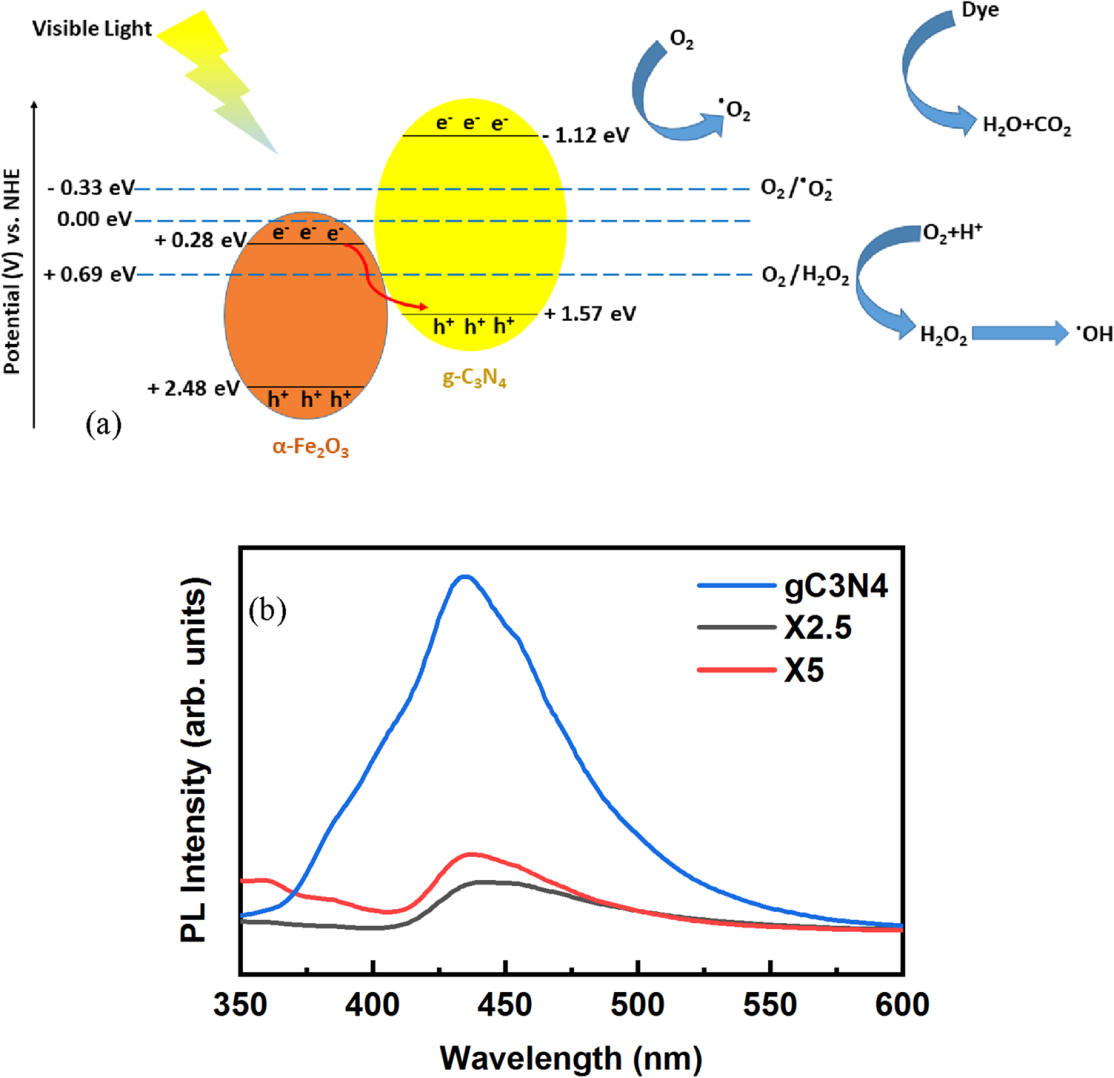
(**a**) Schematic of the band structure and charge transfer mechanism and (**b**) PL spectra of the g-C_3_N_4_, X2.5, and X5 samples.

The charge separation can be described on the base of the band structure between g-C_3_N_4_ and α-Fe_2_O_3_ phases. The position of conduction and valence levels of g-C_3_N_4_ and α-Fe_2_O_3_ can be calculated as follows^25^:

5 $$ E_{CB} = X - 0.5E_{g} - E^{e} $$

6 $$ E_{VB} = E_{g} + E_{CB} $$

which X is the absolute electronegativity (6.90 and 5.83 eV for g-C_3_N_4_ and α-Fe_2_O_3_, respectively), E^e^ is the energy of free electrons on the hydrogen scale (4.5 eV), and Eg is the bandgap energy of the semiconductor, respectively^55^. The positions of E_VB_ and E_CB_ for g-C_3_N_4_ with the bandgap Eg of 2.80 eV are + 1.57 and − 1.12 eV, whereas those are + 2.48 and + 0.28 eV for α-Fe_2_O_3_ with the Eg of 1.9 eV, respectively. The photogenerated charges can be transferred between the various bands of the combined semiconductors in which the electrons go to the less negative CB. In contrast, the holes are reversely transferred to the higher energy level of VB, as schematically shown in Fig. 5a. In fact, the g-C_3_N_4_/α-Fe_2_O_3_/Fe_3_O_4_ nanocomposite follows Z-scheme for charge transfer, because the g-C_3_N_4_ is reduction photocatalyst, while the α-Fe_2_O_3_ is oxidation photocatalyst. In this scheme, the photogenerated electrons with strong reduction abilities in CB of g-C_3_N_4_ and holes with strong oxidation abilities in VB of α-Fe_2_O_3_ are preserved, while the photogenerated electrons in CB of α-Fe_2_O_3_ and holes in VB of g-C_3_N_4_ with inferior redox power recombine^56^. Therefore, the efficient charge separation decreases the recombination rate of electron–hole pairs in the nanocomposites, as can be revealed by PL spectra (Fig. 5b). The X2.5 and X5 composite samples show a weaker PL intensity than pristine g-C_3_N_4_ because of the formation of heterojunction structure between g-C_3_N_4_ and α-Fe_2_O_3_, preventing the electron–hole recombination^57^.

**Figure 6:**
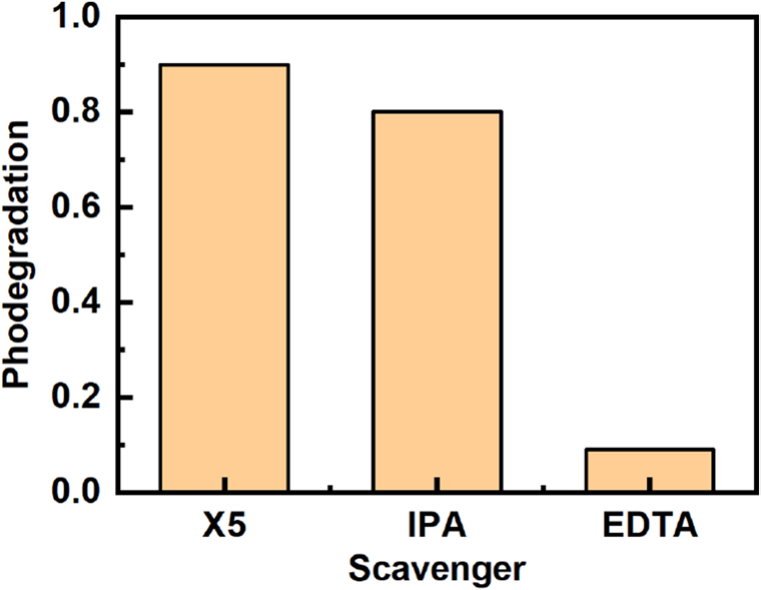
Photodegradation of the MB dye by the X5 composite powders in the presence of a series of scavengers.

Because of the low bandgap energy, the electron–hole pairs are produced on both g-C_3_N_4_ and α-Fe_2_O_3_ under visible-light irradiation. The CB and VB positions of g-C_3_N_4_ are higher than those of the α-Fe_2_O_3_. Some photogenerated electrons on the CB of g-C_3_N_4_ can quickly react with O_2_ to produce ^•^O_2_^−^, because of the more negative CB potential of g-C_3_N_4_ than the potential of the O2/^•^O_2_^−^ (-0.33 eV)^58^. However, the more positive CB potential of α-Fe_2_O_3_ than that of the O_2_/•O_2_- shows that the electrons at CB of α-Fe_2_O_3_ cannot reduce O_2_ to •O_2_-. Moreover, the more negative CB potential of α-Fe_2_O_3_ than the potential of O_2_/H_2_O_2_ (+ 0.682 eV) results in the production of H_2_O_2_ by transferring the accumulated electrons in the CB of α-Fe_2_O_3_ to adsorbed oxygen^59^. The produced H_2_O_2_ molecules can react with the electrons to produce hydroxyl radicals (^•^OH). Furthermore, the photogenerated holes can react with adsorbed H_2_O to produce the hydroxyl radicals (^•^OH) on account of the more positive VB potential than that of •OH/ − OH (+ 2.38 eV)^60^. The photogenerated holes can directly react with the adsorbed dye molecules to produce harmless CO_2_ and H_2_O products. The main reactive species can be realized by using a series of scavengers. Figure 6 shows the photodegradation of MB dye in the presence of 1 mmol disodium ethylenediaminetetracetate (Na_2_-EDTA) and 1 mmol isopropyl alcohol (IPA) for quenching the role of h^+^and ^•^OH radicals, respectively^34^. The decrease of photodegradation in the presence of Na_2_-EDTA is greater than that of IPA. Hence, the effect of photogenerated holes is higher than that of the hydroxyl radicals.

### Electrochemistry analysis

**Figure 7:**
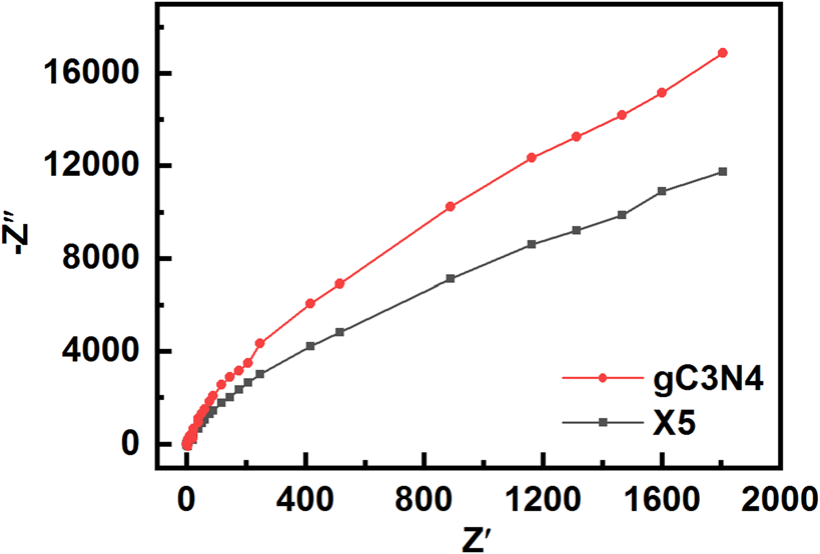
EIS of the pristine g-C_3_N_4_ powders and the X5 composite powders.

The electrochemical impedance spectra (EIS) of the pristine g-C_3_N_4_ and the X5 composite are shown in Fig. 7. The excitation and transfer process of photogenerated charges can be determined from the Nyquist plots. The X5 composite powders have a smaller arc size compared to the pristineg-C_3_N_4_ powders, implying a lower resistance in charge transfer and higher efficiency in charge separation between electron–hole pairs^55^.

**Figure 8:**
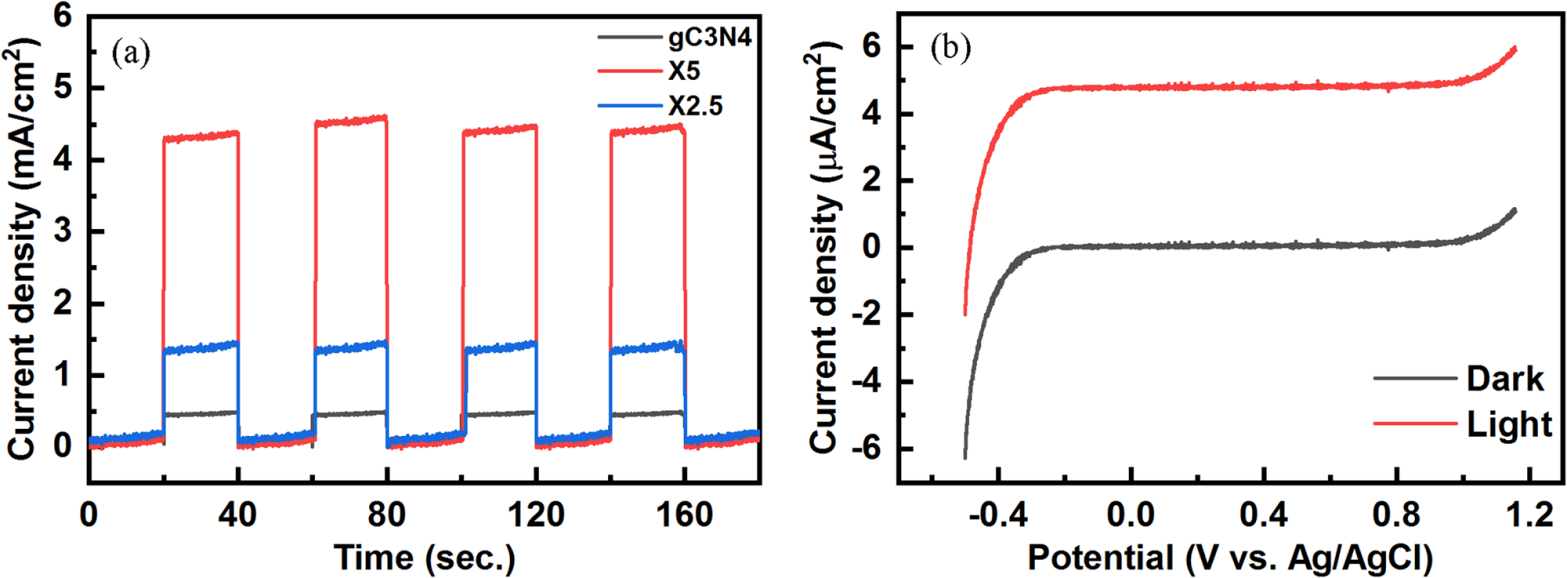
(**a**) Photocurrent versus time under a chopped condition at a constant applied bias of + 0.5 V and (**b**) LSV plot of the X5 composite sample in dark and under visiblelight.

Figure 8a compares the photocurrents produced from the g-C_3_N_4_, X2.5, and X5 samples versus time at a constant applied bias of + 0.5 V. The photocurrent intensity of the X2.5 and X5 composite samples is approximately 3 and 9 times as high as that of the pristine g-C_3_N_4_ powders. Therefore, the photogenerated electrons and holes are effectively separated and easily transferred at the interface of the various semiconductors. Furthermore, the chopping cycles are repeated in each interval time without any changes, showing the photostability of the materials for use in photoelectrochemical cells. Figure 8b illustrates the linear sweep voltammetry (LSV) of the X5 composite sample. A high anodic current is obtained in the presence of visible light irradiation, indicating a facilitated charge transfer to the electrode's surface without significant charge recombination^30^.

## Conclusion

The g-C_3_N_4_/α-Fe_2_O_3_/Fe_3_O_4_ nanocomposites with various types and amounts of iron oxides were successfully synthesized by the solution combustion method. The magnetic properties of the nanocomposites were related to the amount of Fe_3_O_4_ phase, while the optical properties such as absorption coefficient and bandgap energy were dependent on α-Fe_2_O_3_ content. With the combination of 37 wt.% iron oxides to g-C_3_N_4_ (sample X5), the photodegradation of MB, RhB and MO dyes increases from 40 to 75%, from 35 to 58% and from 30 to 53%, respectively, under visible light irradiation. Furthermore, the photocurrent density increased from 0.46 μA/cm^2^ for g-C_3_N_4_ to 4.9 μA/cm^2^ for the α-Fe_2_O_3_/Fe_3_O_4_/g-C_3_N_4_ nanocomposites. The enhancement of photocatalytic performance was recognized by the higher specific surface area, more harvesting of the visible-light irradiation, and efficient separation of the electron–hole pairs.

## Supplementary Information

Supplementary Information.

## Data Availability

The datasets generated during and/or analysed during the current study are available from the corresponding author on reasonable request.
