# Supplementary material for: Effects of iron oxide contents on photocatalytic performance of nanocomposites based on g-C3N4
Source: Sci Rep. 2023 Apr 17;13:6203. doi: 10.1038/s41598-023-33338-1 (PMC10110598; doi:10.1038/s41598-023-33338-1)
Supplement: Supplementary file 1 — Supplementary Information. [file 41598_2023_33338_MOESM1_ESM.docx]

**Supplementary Information**

**Effects of iron oxide contents on photocatalytic performance of nanocomposites based on g-C_3_N_4_**

M. Afkari, S.M. Masoudpanah, M. Hasheminiasari^*^, S.Alamolhoda

*School of Metallurgy & Materials Engineering, Iran University of Science and Technology (IUST),*

*Tehran, Iran*

* Address correspondence to: [mhashemi@iust.ac.ir](mailto:mhashemi@iust.ac.ir)


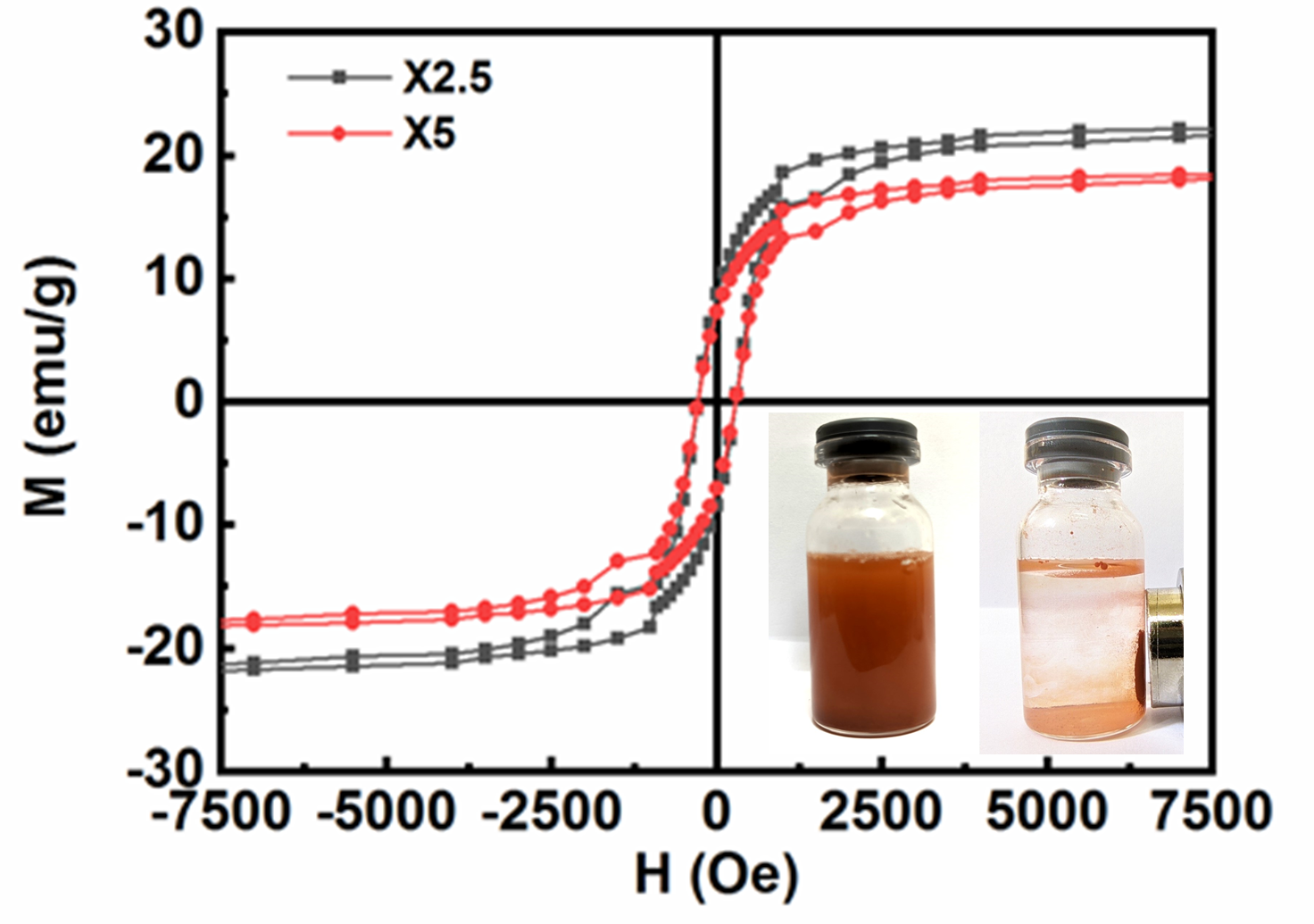


Figure S1. Hysteresis loops of the composite powders and separation of the composite from solution by a magnet.


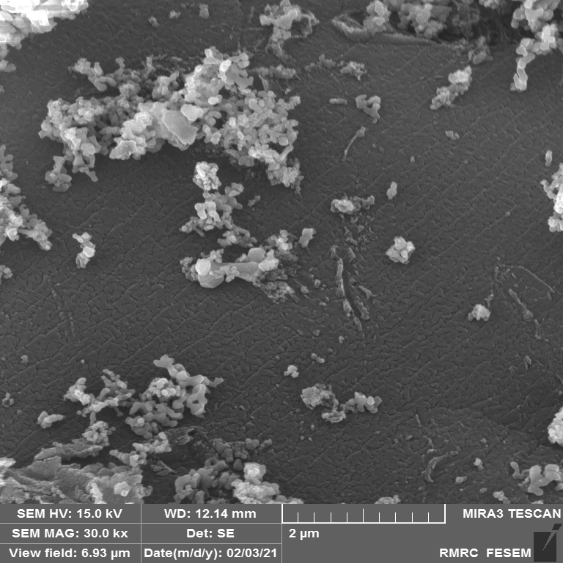

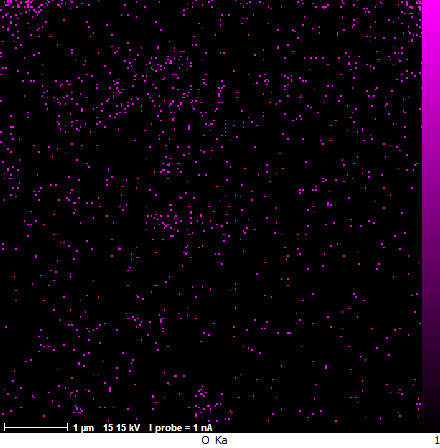

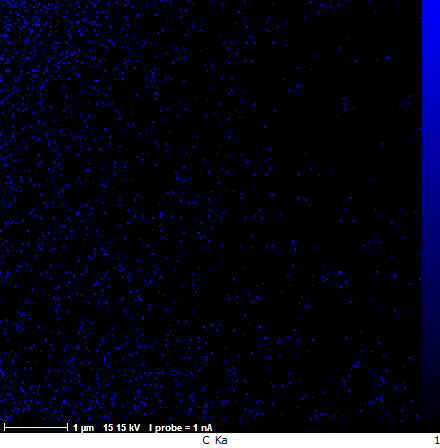


(c)

(b)

(a)


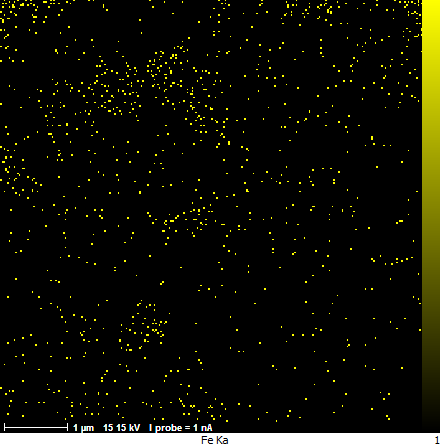

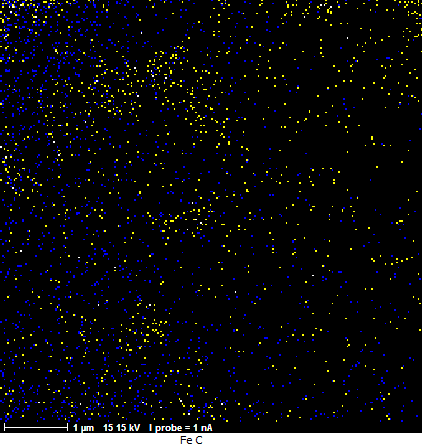


(e)

(d)


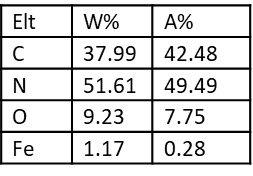


Figure S2. (a) SEM image of X5 powders and elemental distributions of (b) C, (c) O, (d) Fe, and (e) the superposition of the Fe and C elements and the related quantification results.








Figure S3. N_2_ adsorption (filled symbols)-desorption (open symbols) isotherms of the composite samples (The inset shows the pore size distribution plots).







(b)

(a)

Figure S4. (a) UV-Vis diffuse reflectance spectra and (b) Tauc’s plot.


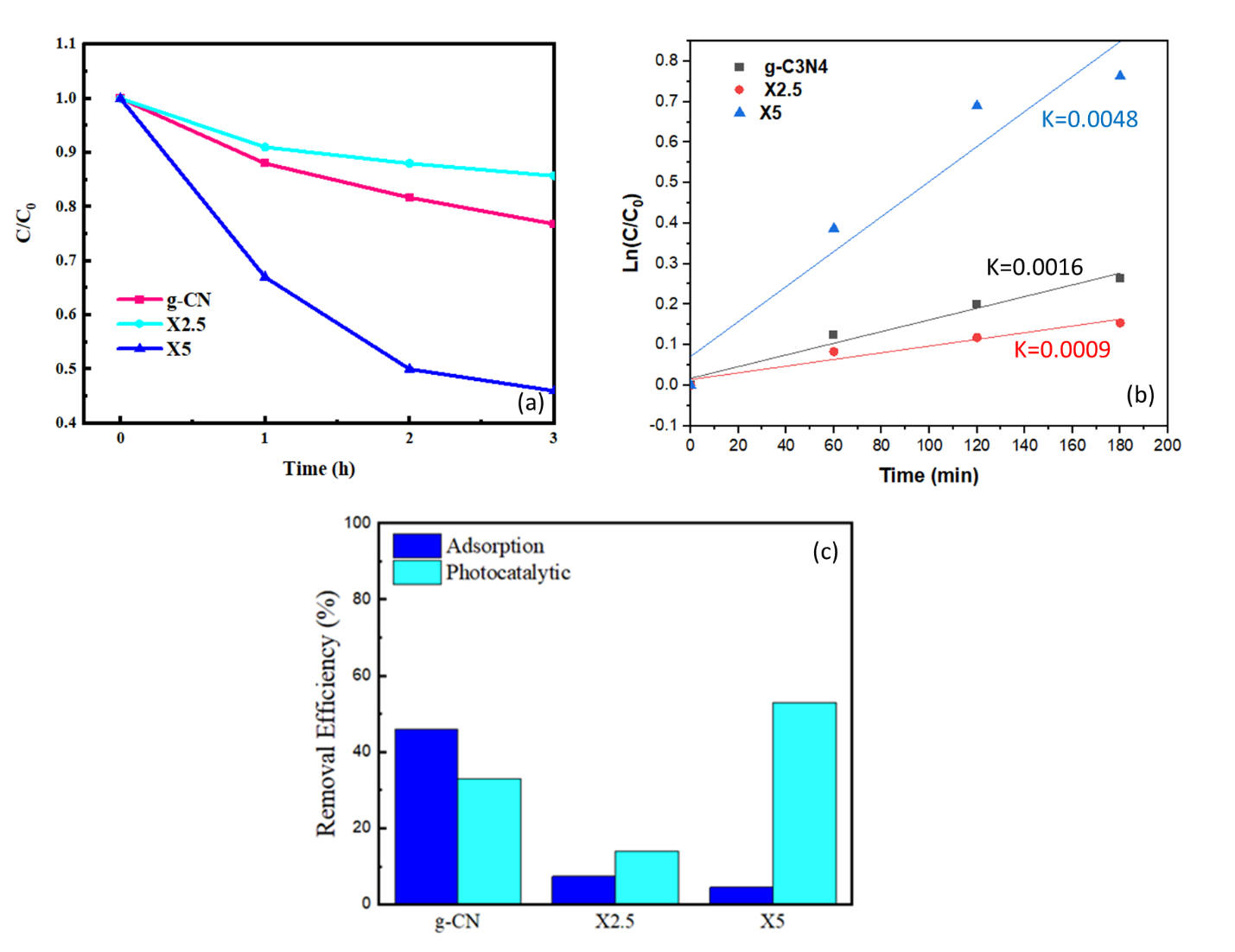


Figure S5. (a) The relative concentration of MO versus illumination time, (b) Ln(C0/C) vs. time for MO and (c) removal efficiency of MO by various catalysts.
